# Bilateral Exudative Retinal Detachment Complicating Preeclampsia With Partial Hemolysis, Elevated Liver Enzymes, and Low Platelet Count Syndrome

**DOI:** 10.7759/cureus.17825

**Published:** 2021-09-08

**Authors:** Mamoun Hani Zebbache

**Affiliations:** 1 Ophthalmology, Central Military Hospital, Kouba, DZA; 2 Faculty of Medicine, University of Algiers, Algiers, DZA

**Keywords:** retinal detachment, hellp syndrome, pre-eclampsia, optical coherence tomography, pregnancy

## Abstract

Serous retinal detachment is an uncommon complication of pregnancy that occurs in well-known situations, such as severe preeclampsia, eclampsia, or hemolysis, elevated liver enzymes, and low platelet count (HELLP) syndrome. The latter still does not reach a consensus, in particular on its classification and pathophysiology. We report the case of a young pregnant woman having a partial HELLP syndrome with massive proteinuria who presented a bilateral exudative retinal detachment having healed spontaneously after pregnancy termination and blood pressure control without requiring an ophthalmologic intervention.

## Introduction

Complicated or even normal pregnancies are associated with ocular changes of varying severity ranging from simple refractive changes to irreversible blindness [1]. These changes can affect the ocular structure and are usually seen as part of pregnancy-related high blood pressure (BP). Exudative retinal detachment (RD) is a rare manifestation of these changes. Exceptionally, pregnancy-induced hypertension is accompanied by neuro-ophthalmologic abnormalities such as cortical blindness or oculomotor disorder due to VI nerve palsy [1].

## Case presentation

We report the case of a 25-year-old primigravida patient who came forward for headaches resistant to usual analgesics. She was then in her 30th week of amenorrhea (29 + 5d). She has an unremarkable medical and ophthalmic history. The pregnancy follow-up was not conducted adequately but we already know that it is a twin pregnancy.

During the initial clinical examination, the patient was conscious, her BP was 160/100 mm Hg and she had pretibial and ankle edema. Several laboratory tests were performed, they revealed abnormal levels of the following parameters: hemoglobin concentration was 9 g/dL (standard: 12-18 g/dL), platelet count was 90 × 103/µL (standard: 130-400 × 103/µL), 24-hour proteinuria was 6.5 g/24 h (standard <300 mg/24 h), and albuminemia was 20.34 g/L (standard: 34-54 g/L). Liver enzymes, as well as bilirubin, blood urea, and serum creatinine, were normal. The obstetric ultrasound revealed an ongoing bi-chorial bi-amniotic twin pregnancy.

The patient was then admitted to the obstetrics department for partial hemolysis, elevated liver enzymes, and low platelet count (pHELLP) syndrome complicating preeclampsia with massive proteinuria [2]. BP was lowered by treatment with methyldopa so that in subsequent measurements BP did not exceed 130/80 mm Hg. The patient was also put on magnesium sulfate as a preventive treatment for eclampsia.

Two days later, the patient reported total blindness upon awakening. This prompted an ophthalmologic examination. Best-corrected visual acuity (BCVA) was then reduced to light perceptions, the anterior segment was unremarkable, and intraocular pressure was within standards. These findings were bilateral.

**Figure 1:**
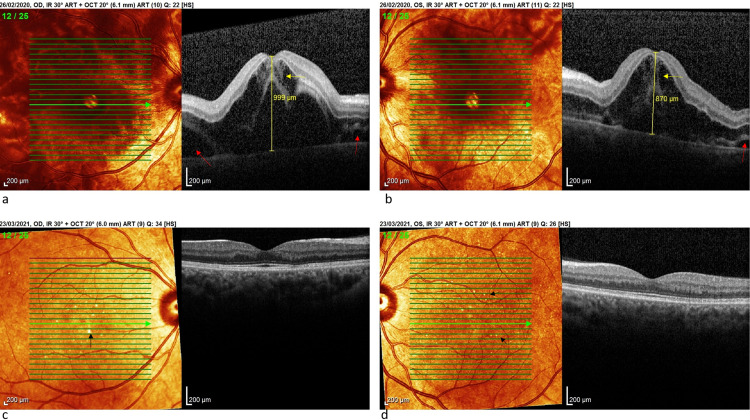
SD-OCT of the macula (a and b) Right eye and left eye, respectively, initial examination showing bilateral macular edema associated with an amount of intra-retinal fluid (yellow arrows) and subretinal fluid (red arrows). (c and d) Examination of both eyes one year after showing the restitution of the retinal anatomy with the persistence of puncture-shaped lesions visible on the infrared image (black arrows). SD-OCT: spectral-domain optical coherence tomography

Dilated fundus examination showed a bilateral bullous serous RD, slightly more significant on the right. No retinal tear was found. Optical coherence tomography (OCT) (Spectralis®, Heidelberg Engineering Inc., Heidelberg, Germany) confirms ophthalmoscopic findings by showing the serous RD involving the macula which had a dome-shaped appearance, central macular thickness being 999µ in the right eye and 870µ in the left (Figure 1). There was a large amount of subretinal and intraretinal fluid.

The patient underwent a cesarean section giving birth to two premature twins of different sexes. After a stay in neonatal intensive care, the newborn male died.

**Figure 2:**
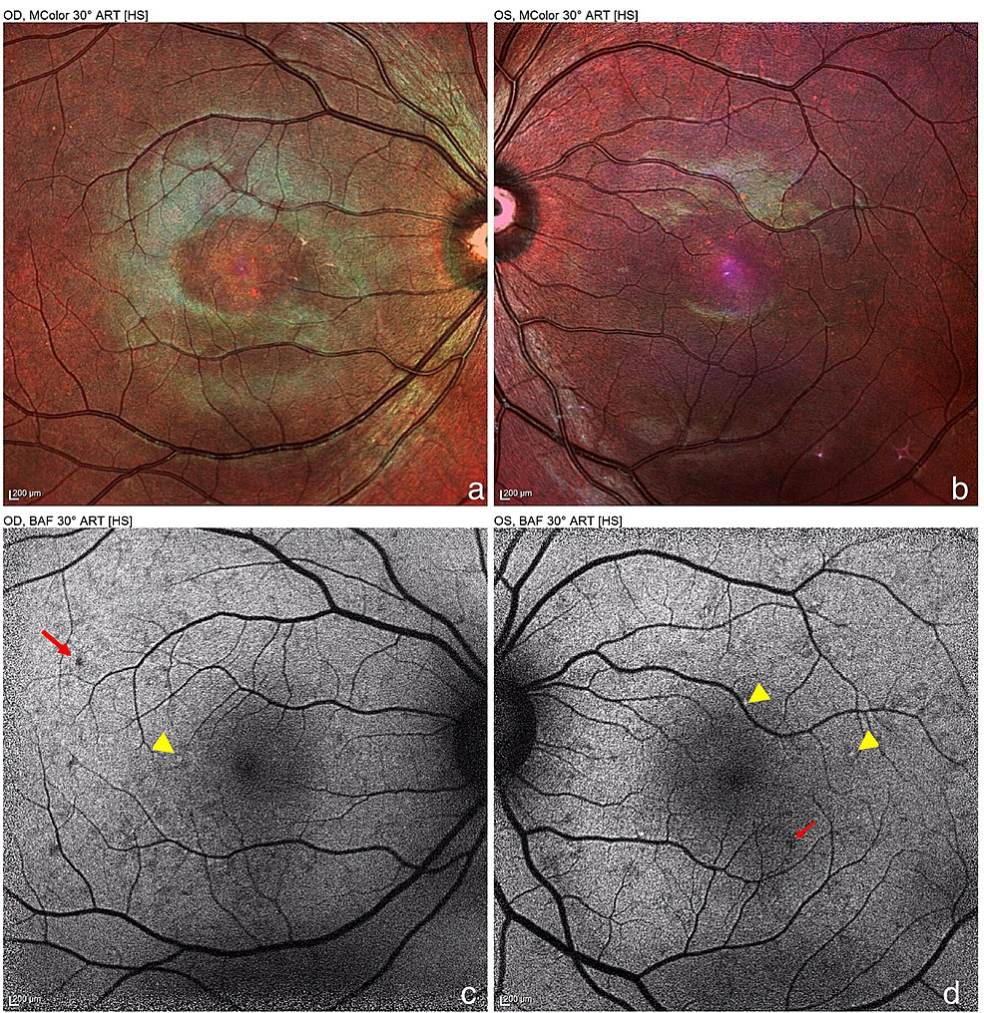
MC image (a and b) and FAF (c and d) one year after the initial episode MC shows a mottled aspect of the RPE. On FAF, some spots are hyperautofluorescent (yellow arrowheads), others have no translation. We note the presence of hypoautofluorescent spots (red arrows) which are not visible on the MC image. MC: multicolor; RPE: retinal pigment epithelium; FAF: fundus autofluorescence

She did not return to our consultation after discharge from the hospital and was not seen again until a year later. She reports that she had felt an improvement in visual acuity a few days after delivery. BCVA is now at 20/20 in both eyes. Examination of the fundus finds a few small yellowish punctiform formations scattered around the posterior pole (Figure 2). OCT finds a normal macular profile but the retinal pigment epithelium (RPE) has small growths at the level of yellowish spots observed ophthalmoscopically (Figure 1).

## Discussion

HELLP syndrome is a complication of pregnancy characterized by three criteria: hemolysis, elevated liver enzymes, and low platelet count. Partial HELLP syndrome (pHELLP) is defined by the presence of one or two features of HELLP syndrome but not the complete form [2-4]. There are no reliable data on its incidence [3].

Preeclampsia is a multisystemic disease occurring in 3-5% of pregnancies in the third trimester, classically characterized by a new-onset high BP (systolic BP ≥ 140 mm Hg and/or diastolic BP ≥ 90 mmHg) accompanied by proteinuria [5].

HELLP syndrome represents a severe form of preeclampsia [6]; however, the diagnosis of HELLP syndrome can be made without underlying preeclampsia [7]. Subjective visual symptoms are reported by approximately 40% of preeclamptic patients but blindness is rare. Funduscopic examination usually shows localized or diffuse arterial narrowing or even classic signs of hypertensive retinopathy [1]. RD is an unusual situation in pregnant women, it affects 0.1-2% of patients with severe preeclampsia and 0.9% of patients with HELLP syndrome. It usually affects primiparous women and is seen in the third trimester or shortly after delivery [6]. Exudative RD results from the rupture of the outer blood-retinal barrier (oBRB) formed by tight junctions between the cells of the RPE, one of the major functions of which is the maintenance of the subretinal space in the virtual state by transporting the liquid out of it.

The pathophysiology of HELLP syndrome is believed to be similar to that of disseminated intravascular coagulation (DIC). Thus, microthrombi that are formed disrupt choroidal circulation [7]. The additional combination of arterial hypertension and nephropathy contributes to aggravate this situation [6]. The resulting hypoxic damage to the oBRB results in significant failure of this barrier, as evidenced by the bullous nature of RD. In addition, hypoalbuminemia also contributes to the formation of RD [6]. Massive proteinuria (rate greater than 5 g/24 h) would be associated with a higher incidence of RD compared to lower proteinuria levels [5].

OCT is a non-invasive, informative, and reproducible retinal imaging technique, it provides an instantaneous and quasi-histological image of the retina. Unlike fluorescein angiography (FA) theoretically considered as potentially teratogenic, OCT is safe in pregnant women regardless of gestational age [8]. This technique could confirm ophthalmoscopic findings and detect conditions that may escape clinical examination. OCT is also used to measure choroidal thickness. In a report, this choroidal thickness was significantly increased in patients with serous RD compared to other patients with preeclampsia [9]. OCT should be performed if possible in all pregnant women with preeclampsia/eclampsia or HELLP syndrome, especially if they have visual complaints.

FA could be carried out in women affected postpartum; in these cases, the investigation revealed severe choroidal damage, which supports the pathophysiological hypotheses [10]. Although the cause of blindness seems obvious, some authors perform brain imaging to detect any associated abnormalities [10].

There are no explicit recommendations that would guide the management of exudative RD in the context of preeclampsia, but it is advisable to terminate the pregnancy by cesarean section as soon as possible [11]. The anatomical and functional sequelae of RD in this setting are usually mild. Residual lesions usually appear as mottling of the fundus, there are not much data available on their ophthalmoscopic, angiographic, or fundus autofluorescence features. Blindness can result from cortical damage or retinal arterial occlusions. Nevertheless, one publication reported total blindness following exudative RD, however, this report only specifies low visual acuity and does not describe the appearance of the fundus [10].

Extensive studies of the retinal microcirculation that is accessible to the direct visual examination in vivo could provide pieces of information on the state of the uteroplacental microvasculature during high-risk pregnancies [12].

## Conclusions

Fortunately, RD associated with preeclampsia does not progress on its own and returns to order with the correction of systemic disorders. Nonetheless, this does not exempt mandatory ophthalmic examination with appropriate follow-up in any pregnant woman with visual complaints.
